# Twenty years of experience of a tertiary cancer center in total body irradiation with focus on oncological outcome and secondary malignancies

**DOI:** 10.1007/s00066-022-01914-5

**Published:** 2022-03-22

**Authors:** Katharina Sieker, Maximilian Fleischmann, Martin Trommel, Ulla Ramm, Jörg Licher, Gesine Bug, Hans Martin, Hubert Serve, Claus Rödel, Panagiotis Balermpas

**Affiliations:** 1 Department of Radiation Oncology, University Hospital—Goethe University, Theodor-Stern-Kai 7, 60590 Frankfurt am Main, Germany; 2 Department of Medicine 2, Hematology/Oncology, Goethe University, Frankfurt, Germany; 3 Frankfurt Cancer Institute, Goethe University, Frankfurt/Main, Germany; 4 Department of Radiation Oncology, University Hospital Zurich, Zurich, Switzerland; 5 German Cancer Research Center (DKFZ), Heidelberg, Germany; 6 German Cancer Consortium (DKTK) partner site: Frankfurt am Main, Frankfurt am Main, Germany

**Keywords:** TBI, Sequelae, Toxicity, Long-term follow-up, Hematopoietic stem cell transplant

## Abstract

**Purpose:**

Total body irradiation (TBI) is a common part of the myelo- and immuno-ablative conditioning regimen prior to an allogeneic hematopoietic stem cell transplantation (allo-HSCT). Due to concerns regarding acute and long-term complications, there is currently a decline in otherwise successfully established TBI-based conditioning regimens. Here we present an analysis of patient and treatment data with focus on survival and long-term toxicity.

**Methods:**

Patients with hematologic diseases who received TBI as part of their conditioning regimen prior to allo-HSCT at Frankfurt University Hospital between 1997 and 2015 were identified and retrospectively analyzed.

**Results:**

In all, 285 patients with a median age of 45 years were identified. Median radiotherapy dose applied was 10.5 Gy. Overall survival at 1, 2, 5, and 10 years was 72.6, 64.6, 54.4, and 51.6%, respectively. Median follow-up of patients alive was 102 months. The cumulative incidence of secondary malignancies was 12.3% (*n* = 35), with hematologic malignancies and skin cancer predominating. A TBI dose ≥ 8 Gy resulted in significantly improved event-free (*p* = 0.030) and overall survival (*p* = 0.025), whereas a total dose ≤ 8 Gy and acute myeloid leukemia (AML) diagnosis were associated with significantly increased rates of secondary malignancies (*p* = 0.003, *p* = 0.048) in univariate analysis. No significant correlation was observed between impaired renal or pulmonary function and TBI dose.

**Conclusion:**

TBI remains an effective and well-established treatment, associated with distinct late-toxicity. However, in the present study we cannot confirm a dose–response relationship in intermediate dose ranges. Survival, occurrence of secondary malignancies, and late toxicities appear to be subject to substantial confounding in this context.

**Supplementary Information:**

The online version of this article (10.1007/s00066-022-01914-5) contains supplementary material, which is available to authorized users.

## Introduction

Total body irradiation (TBI) is a common and effective part of the myelo- and immuno-ablative conditioning regimen prior to an allogeneic hematopoietic stem cell transplantation (allo-HSCT) for patients with malignant and nonmalignant hematologic diseases [1]. Since its introduction by Nobel Prize Laureate E. D. Thomas in the late 1950s [2, 3], TBI concepts have evolved [4]. Early treatment regimens combined a lethal dose of TBI with or without cyclophosphamide (Cy) to eradicate cancer cells in patients with acute myeloid leukemia (AML) [5]. Although, improvements regarding technical aspects and fractionation have reduced acute and late toxicities [6, 7], TBI is associated with immediate and long-term posttransplant complications which can impair both quality of life and outcome [8–10]. In general, complications after allo-HSCT are multifactorial, but there is a broad spectrum of particularly radiation-related side effects including cataract, endocrine and gonadal insufficiency, renal malfunction, lung fibrosis, cardiomyopathy, veno-occlusive disease (VOD) and others [11, 12]. Moreover, the development of secondary malignancies is a serious long-term toxicity, caused by extensive radiation and chemotherapy as well as the profound immunosuppression after allo-HSCT [13–16]. As a consequence, alternative conditioning protocols were pursued, and reduced-intensity and chemotherapy-based regimens were implemented [17–19]. Further improvements in donor selection and high-resolution human leukocyte antigen (HLA) matching, as well as management of infections posttransplant, prevention and treatment of graft-versus-host disease (GVHD) have ameliorated treatment-related mortality and morbidity significantly [20, 21]. Accordingly, chemotherapy-based and reduced-intensity conditioning (RIC) regimens have become increasingly well accepted and are substituting otherwise successfully established TBI-based treatment regimens due to general concerns regarding tolerability and toxicity [22, 23].

These developments resulted in a constant decline in the application of TBI as a first-line treatment modality. Nevertheless, contradictory results from previous analyses leave the question of the optimal conditioning regimen unanswered, while an increasing number of patients qualifying for an intensified therapy protocol after relapse [24–26]. Endpoints of this study are the long-term survival and the incidence of late toxicity, including secondary malignancies, after TBI-based conditioning regimens of various intensities prior to allo-HSCT in a single-center cohort of 285 patients.

## Patients and methods

### Patients—inclusion criteria

We performed a retrospective analysis of patients with hematologic diseases, who underwent a TBI as part of the myelo- and immune-ablative conditioning regimen prior to allo-HSCT at the University Hospital Frankfurt between 1997 and December 2015. Patients had to be 18 years or older at the time of irradiation and received at least one fraction of TBI. Adolescents (≥ 16 years) treated in analogy to protocols for adult patients were also included. The choice of conditioning regimen was made by the treating physicians according to the respective standard of care, within a clinical trial, or after an interdisciplinary tumor conference. Data on disease control and toxicity were retrieved from the patient files. In addition, patients alive were also contacted and asked to complete a specific questionnaire including questions about general health status, comorbidities and any therapy-associated malignancies according to organ systems. The study was approved by the institutional review board (approval number 221/16).

### Radiotherapy—conditioning chemotherapy regimens

All radiotherapy series were applied with linear accelerators, using exclusively 6 MV energy photons and implementing a motorized patient translational couch (Barry-Liege®, Jochen Barry GmbH, Essen, Germany) with variable, preselectable transport speed moving the patient through an open radiation field. The radiation field measures 15 cm in length and 40 cm in width at the isocenter. TBI was performed in two daily fractions of 1.8 Gy (1997–2003) or 2 Gy (2003 until today), administered at least 6 h apart. The patients were irradiated alternating in prone and supine position for each Gray (Gy) of dose applied. A tissue-equivalent bolus in the neck region is used to reduce the risk of overdose due to a smaller diameter. In addition, a Plexiglas® (Röhm GmbH, Darmstadt, Germany) (polymethylmethacrylate glass) bolus was used to cover the entire field below the eyes to increase the skin dose. In vivo dosimetry with semiconducting diodes was performed for dose verification. Six semiconducting diodes were placed under the eye, on the neck behind the bolus, behind the ventral lung block, in the dorsal lung region, near the umbilicus (i.e., the ventral reference point) and its dorsal counterpart (i.e., the dorsal reference point). The detailed procedure was previously described by Ramm et al. in 2008 [27]. Customized, partial lung shields were used for prescribed doses > 10 Gy.

For patients with acute leukemia, treatment regimens typically included a remission induction chemotherapy followed by a conditioning regimen in preparation for allo-HSCT. Standard remission induction chemotherapy for patients with acute myeloid leukemia (AML) consisted of one or two courses of cytarabine and anthracycline (7 + 3) [28]. After induction chemotherapy, allo-HSCT was recommended for (1) suitable patients in first complete remission with intermediate- or high-risk disease determined by cytogenetic or molecular genetic abnormalities and (2) for patients with relapsed disease [29, 30]. Patients with acute lymphoblastic leukemia (ALL) were treated within or according to the respective protocols of the GMALL study group and typically transplanted after the first consolidation cycle [31].

After completion of induction chemotherapy, the conditioning regimen was evaluated. These are fixed combinations adapted to the patient and risk factors with a characteristic profile of adverse events and toxicities. For patients with AML or ALL in complete remission up to the age of 40–50 years, the most frequently used myeloablative conditioning (MAC) regimen consisted of 12 Gy TBI in combination with cyclophosphamide or etoposide. Patients above the age of 40 or with comorbidities were eligible for a reduced intensity conditioning (RIC) regimen of 8 Gy TBI and fludarabine. In case of active disease, sequential conditioning regimens were used such as melphalan followed by 8 Gy TBI and fludarabine for AML or fludarabine with amsacrine and cytarabine (FLAMSA) followed by 12 Gy TBI for ALL, respectively. Prophylaxis for graft-versus-host disease and supportive care including anti-infective prophylaxis with antibiotics, anti-viral and anti-fungal drugs, growth factors and transfusion were provided according to standard of care procedures.

### Follow-up and toxicity assessment

Follow-up procedures were scheduled according to national and international recommendations. To ensure an extensive posttransplant care, multidisciplinary long-term follow-up was initiated 12 months after allo-HSCT and successful engraftment. Follow-up procedures included graft-versus-host disease (GVHD) screening, serum chemistry, including thyroid function testing (thyroid-stimulating hormone levels [TSH], free triiodothyronine [fT3], and thyroxine [T4]), screening for hypogonadism (in men: baseline luteinizing hormone [LH] and testosterone levels; in women: baseline follicle stimulating hormone [FSH], LH and 17β-estradiol levels) and cortisol levels if necessary, assessment of liver function (bilirubin, aspartate aminotransferase [AST], alanine aminotransferase [ALT], alkaline phosphatase [ALP], hepatitis c virus [HCV] and hepatitis B virus [HBV] screening), iron metabolism (serum iron, ferritin, transferrin, haptoglobin, folic acid, vitamin B12, total iron binding capacity, transferrin saturation, soluble transferrin receptor), triglycerides, high-density lipoprotein (HDL) and low-density lipoprotein (LDL) levels. Blood count including lymphocyte subpopulations and reticulocytes. Evaluation of renal function with serum creatinine, blood urea nitrogen, and 24 h urine analysis including creatinine clearance, total protein, albumin and electrolytes.

Remission control is performed via bone marrow examination including chimerism monitoring and measurement of minimal residual disease (MRD). Computed tomography (CT) scans were performed as necessary.

Instrumental diagnostics include an electrocardiogram, echocardiogram, pulmonary function test, and diffusion capacity as well as baseline bone density testing.

Ophthalmologic examination includes assessment of visual acuity, fundoscopic examination and intraocular pressure measurement. Regular dermatological inspections are obligatory. Furthermore, dental health care, smoking cessation and vaccinations are recommended.

Follow-up examinations were initially performed every 3 months and later every 6 or 12 months, or according to individual risk. Toxicity was evaluated according to the Common Terminology Criteria for Adverse Events (CTCAE) version 5.0.

### Statistical analyses

In this retrospective analysis, we used Spearman’s correlation coefficient for the assessment of the association between the variables. Time-to-event data were plotted according to the Kaplan–Meier method, while significance was calculated using the log-rank test. Overall survival was defined as the time from first fraction of radiotherapy to death from any cause or last follow-up. Event-free survival was defined as time from first radiotherapy to death from any cause, recurrence of the hematological disease or any other malignancy. The predefined primary endpoint of the study was event-free survival; secondary endpoints were overall survival, the cumulative incidence of secondary malignancies with death as competing risk and descriptive analyses and correlations of the above with patient and treatment characteristics. Impaired organ function was assessed using common parameters such as creatinine clearance, liver function parameters, or pulmonary function tests.

All tests were two sided and a *p*-value of 0.05 was considered statistically significant. The SPSS (version 25, IBM, Armonk, NY, USA) was used to perform all statistical analyses.

## Results

### Patient and treatment characteristics

We identified a total of 285 patients (female, *n* = 124, 43.5%) with hematopoietic diseases with a median age of 45 years (range: 16–65), who underwent a TBI as an integral part of the myelo- and immuno-ablative conditioning regimen followed by subsequent allo-HSCT at our institution between 1997 and 2015. The median follow-up for all patients was 49 months (range, 0–260 months) and 102 months (range, 36–251 months) for all patients alive. Patients were treated with allo-HSCT for acute myeloid leukemia (AML, *n* = 146), acute lymphoblastic leukemia (ALL, *n* = 91), lymphomas (*n* = 25) or other diseases as myelodysplastic syndrome or aplastic anemia (*n* = 23). The median radiotherapy dose applied was 10.5 Gy (range: 1.9–13.5 Gy, measured with in vivo dosimetry), with the most common dose of 8 Gy for AML and 12 Gy for ALL. The most commonly prescribed TBI dose was 12 Gy (*n* = 141, 49.5%) followed by 8 Gy (*n* = 90, 31.6%) and 4 Gy (*n* = 33, 11.6%). In all, 14 (4.9%) patients discontinued an ongoing series/TBI due to complications. In all, 238 patients (83.5%) received a total dose of at least 8 Gy. All characteristics are summarized in Table 1.

**Table 1:** Patient, disease and treatment characteristics

		Patients, *n*	%
*Gender*			
Male	–	161	56.5
Female		124	43.5
*Age*			
Median	45 years	–	–
Range	16–65 years	–	–
< 45 years		137	48.1
≥ 45 years		148	51.9
*Diagnosis*			
AML	–	146	51.2
ALL		91	31.9
Lymphoma		25	8.8
Other		23	8.1
*TBI dose*			
Median	10.5 Gy	–	–
Range	1.9–13.5 Gy	–	–
4 Gy		33	11.6
8 Gy		90	31.6
12 Gy		141	49.5
Other (> 8 Gy)		7	2.5
Discontinued		14	4.9
*Recurrence of primary disease*			
No	–	196	68.8
Yes		89	31.2
*Secondary Malignancy*			
No	–	250	87.7
Yes		35	12.3

*AML* acute myeloid leukemia, *ALL* cute lymphoblastic leukemia, *TBI* total body irradiation

### Clinical outcomes: disease control and survival

**Fig. 1:**
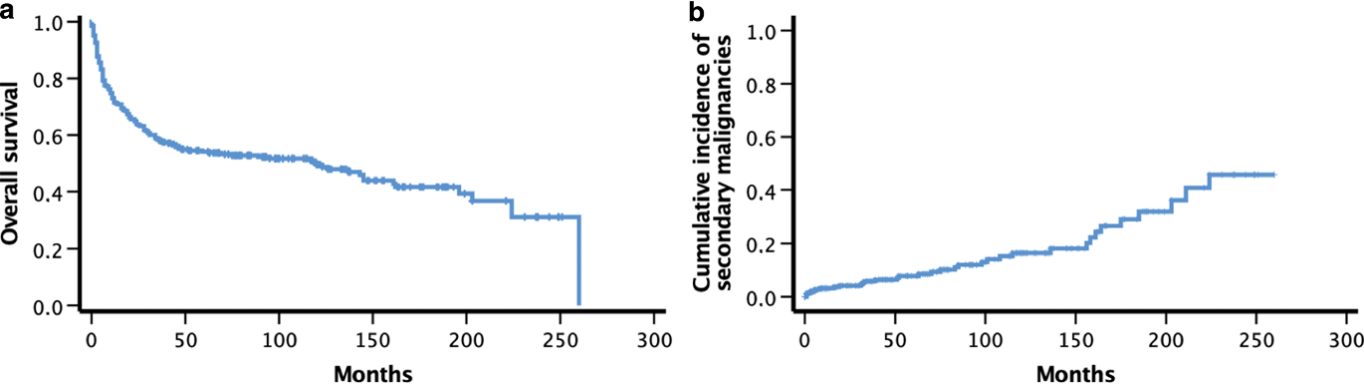
Overall survival of all patients (**a**) and cumulative incidence of secondary malignancies during the observation period (**b**). *ALL* acute lymphoblastic leukemia, *AML* acute myeloid leukemia

Overall survival (OS) at 1, 2, 5 and 10 years was 72.6, 64.6, 54.4 and 51.6%, respectively. The median OS for all patients was 120 months (Fig. 1). A total of 151 (52.9%) patients died during the observation period including 100 patients due to recurrence or progression of the underlying disease (66.2% of all deaths), 8 patients due to infections (5.3%), 5 patients due to acute GVHD (3.3%) and 38 patients following other transplant-related events (25.1%). Two patients died from unknown causes. This results in a cumulative incidence of treatment-related mortality of 17.9% for the complete cohort. Sex (*p* = 0.669) and age (*p* = 0.188) had no impact on survival in our cohort. Patients treated with TBI for the diagnoses of ALL (*n* = 91), AML (*n* = 146) or other hematological diseases (*n* = 48) had a comparable OS without significant differences (*p* = 0.559). In all, 89 patients (31.2%) had recurrent or refractory disease after allo-HSCT, of whom 46 patients relapsed in the first 24 months at a median time of 7 months (range 2–24). Furthermore, 56 events were reported in the first 6 months, 82 within 12 months, and 105 within 24 months. After 5 years, 142 events were recorded.

**Fig. 2:**
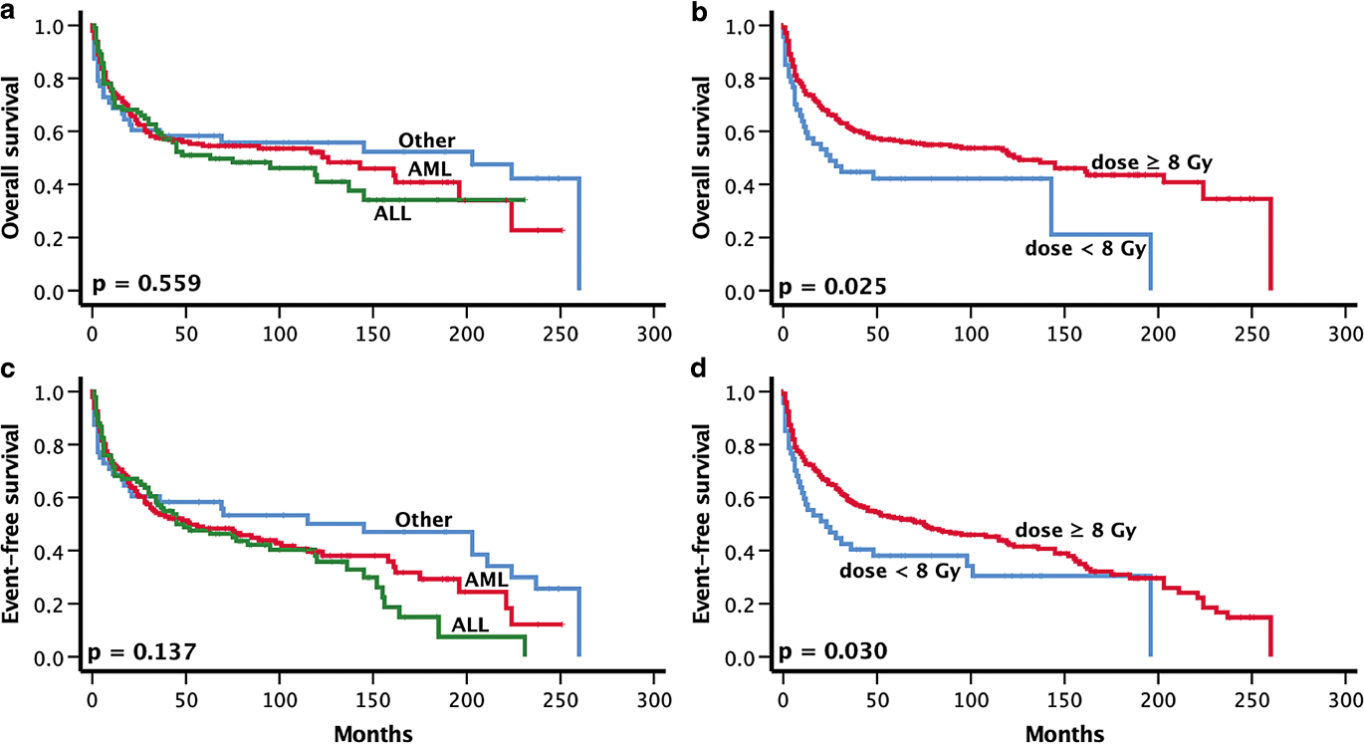
Overall survival and event-free survival according to primary disease (**a**, **c**) and applicated dose (**b**, **d**). *ALL* acute lymphoblastic leukemia, *AML* acute myeloid leukemia

Patients who received a dose of ≥ 8 Gy had a superior overall (*p* = 0.025) and event-free survival (*p* = 0.030), respectively. There was no further significance between radiation-dose and overall survival for higher dose cut-offs. Altogether, significantly less events (any death, recurrent disease or secondary malignancies) were reported in the group of patients who received at least 8 Gy (*p* = 0.03; Fig. 2).

### Secondary malignancies

**Fig. 3:**
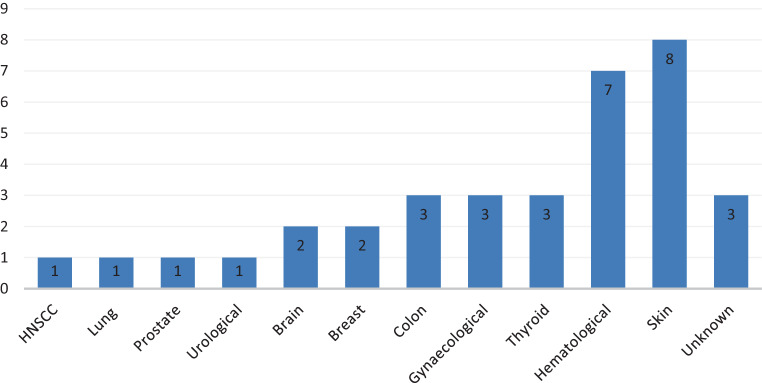
The frequency of secondary malignancies in our cohort of patients who underwent a total body irradiation as a part of their conditioning regimen in prior to hematopoietic stem cell transplantation (HSCT). *HNSCC* head and neck squamous cell carcinoma

**Fig. 4:**
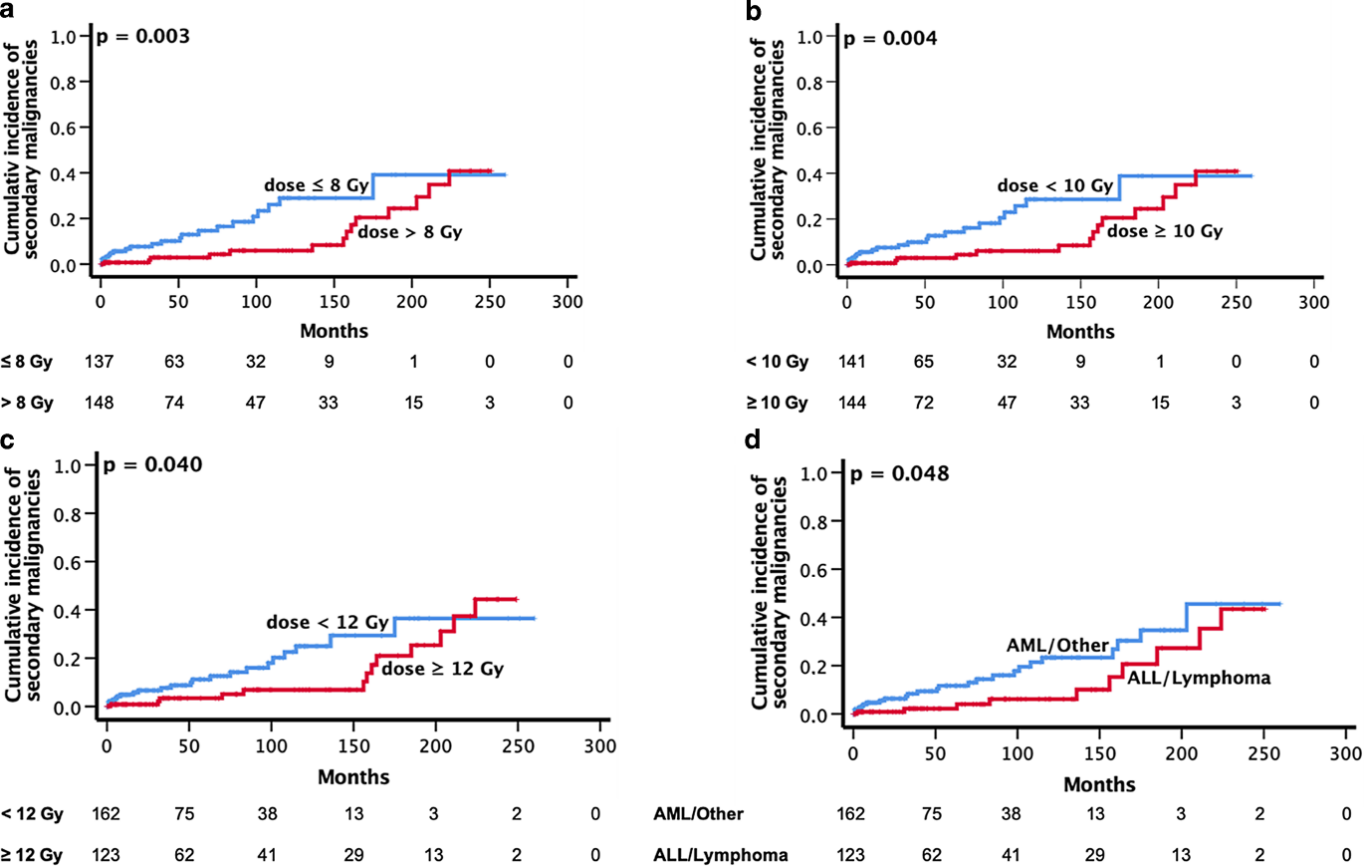
Cumulative incidence of secondary malignancies stratified by total dose (**a**, **b** and **c**) and by primary disease (**d**). The numbers of patients at risk are given below. *ALL* acute lymphoblastic leukemia, *AML* acute myeloid leukemia

Thirty-five secondary malignancies occurred in 33 patients after 7–224 months (Fig. 1). The cumulative incidence of secondary malignancies for all patients after 5 and 10 years was 5.6 and 8.8%, respectively. Of the 35 secondary malignancies, 28 were solid and 7 were hematologic. Two patients developed more than one secondary malignancy (nonmelanoma skin cancer and colorectal cancer or gynecological cancer, respectively). Skin cancer (*n* = 8) and hematological malignancies (*n* = 7) occurred most frequently followed by thyroid (*n* = 3), colon (*n* = 3) and gynecological cancers (*n* = 3). A detailed overview of the occurring secondary malignancies is given in Fig. 3. Seven patients died because of secondary malignancies after a median time of 10 months (range 1–64 months). In this cohort, there was no relationship between the risk of secondary malignancies and sex or age at different cut offs (median and 60 years) (*p* = 0.809, *p* = 0.932 and *p* = 0.546, respectively). After univariate analysis, the risk of developing a secondary malignancy and the OS among patients undergoing a TBI were both associated with the total dose of irradiation: lower doses (cut-off ≤ 8 Gy, < 10 Gy and < 12 Gy) were significantly correlated with an increased risk of secondary malignancies (*p* = 0.003, *p* = 0.004 and *p* = 0.040, respectively). Patients diagnosed with AML or other myeloid diseases had also an increased risk of secondary malignancies (*p* = 0.048) when compared to patients with ALL or lymphoma (Fig. 4).

After multivariate analysis, no significance remained. A summary of the significant values according to the univariate and the corresponding multivariate analyses for the most important time-to-event endpoints can be found in Table 2.

**Table 2:** Univariate and multivariate analyses. Significant values are given in bold

			Multivariate analyses		
			95% Confidence interval		
	Univariate *p*-value	HR	Lower	Upper	*p*-value
*Overall survival*			–	–	–
Dose (</≥ 8 Gy)	**0.025**	1.580			
Event-free survival			–	–	–
Dose (</≥ 8 Gy)	**0.030**	0.657			
*Cumulative incidence of secondary malignancies*					
Dose (≤/> 8 Gy)	**0.003**	1.495	0.966	2.315	0.071
Dose (</≥ 10 Gy)	**0.004**	0.859	0.463	1.594	0.630
Dose (</≥ 12 Gy)	**0.040**	1.227	0.700	2.151	0.474
AML/other vs. ALL/lymphoma	**0.048**	1.012	0.725	1.413	0.945

*ALL* acute lymphoblastic leukemia, *AML* acute myeloid leukemia, *HR* hazard ratio

### Other toxicity

**Fig. 5:**
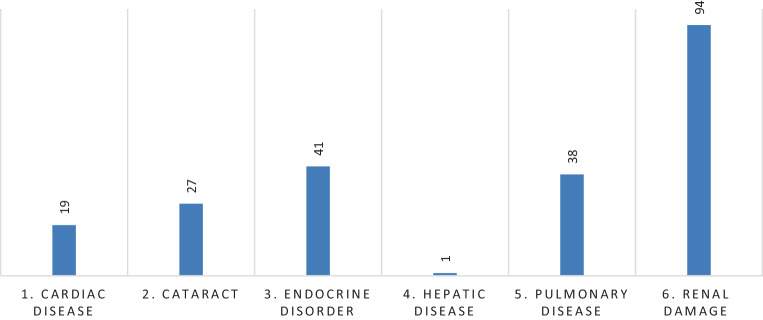
Total number of chronic disorders after treatment

In all, 220 nonmalignant chronic morbidities were reported as depicted in Fig. 5. In total, 144 patients developed severe organ damage (50.5%). Most prevalent nonmalignant late toxicity was decline in renal function in 33% of the patients, followed by endocrine disorders in 14.4% and a decline in pulmonary function in 13.3%. No significant correlation was observed between the impairment of renal and pulmonary function and increasing TBI dose. Any acute toxicity including acute GVHD grade > II requiring systemic treatment, was reported in 92 cases (32.3%; supplementary Tables 1 and 2).

## Discussion

The present study presents epidemiological data, oncological outcome and late toxicity of a large cohort treated with TBI in a tertiary cancer center. While overall survival exceeded 50% at 10 years, we report considerable rates of sequelae, including secondary malignancies. These late effects did not seem to be correlated with higher irradiation doses in an intermediate dose range (6–12 Gy), while higher TBI doses were significantly correlated with improved outcomes. Therefore, overall and event-free survival were significantly higher in patients receiving at least 8 Gy total dose (*p* = 0.025 and *p* = 0.030) without further improvements for any dose above.

Based on the limited number of studies, most of which are underpowered and involve small and heterogeneous cohorts, a conflicting and inconsistent literature emerges, leaving the optimal conditioning regimen elusive [32–34]. Combining two modalities is a common approach in oncology. Overall, there is a growing body of evidence of using reduced-intensity and chemotherapy-based conditioning regimens to increase overall survival and reduce treatment-related mortality. After reduced-intensity conditioning regimens, a higher relapse rate has been reported in general, while overall survival increased due to a significant decrease in treatment-related mortality [35]. The only prospective study (BMT CTN 0901) comparing myeloablative versus reduced-intensity, chemotherapy-based conditioning regimens has been closed due to a higher relapse rate and only a modest decrease in treatment-related mortality in the reduced-intensity arm [36]. Although only 8 of 272 AML or MDS patients underwent a myeloablative conditioning regimen including total body irradiation, the study supports the use of myeloablative conditioning regimens for patients who are able to tolerate this intensified treatment [36].

Another prospective, open label randomized phase III trial compared an 8 Gy TBI and fludarabine-based conditioning regimen with a 12 Gy TBI and cyclophosphamide-based conditioning regimen. Within the 8 Gy TBI + Cy cohort, there was a reduced acute toxicity with no change the in incidence of nonrelapse mortality and survival [37]. Long-term results revealed that the reduced-intensity conditioning regime with moderately reduced total body irradiation doses was associated with lower acute morbidity and toxicity, while the risk of late relapse remained unaffected. Notably, the study has failed the recruitment and was closed early [38].

Therefore, reduced-intensity conditioning regimens, especially for older patients, became predominate [39]. Malard et al. compared two cohorts receiving allo-HSCT between 1983 and 2010. Patients in the cohort between 2001 and 2010 were older and reduced-intensity conditioning became more prevalent. Overall survival was improved by a significantly decreased nonrelapse mortality, while relapse rate was increased [40]. These results strengthen the aforementioned assumption, while this could further be a result of improvements in donor selection like high-resolution HLA matching as well as supportive care and thereby reducing the risk of posttransplant complications [40, 41].

In another retrospective cohort study, Gooptu et al. evaluated the outcome of 387 patients undergoing a myeloablative conditioning regimen consisting of cyclophosphamide and TBI (CyTBI) versus fludarabine and busulfan (FluBu). Again, mediated by significantly lower treatment-related mortality and similar relapse rates, a significantly improved overall survival was demonstrated in the chemotherapy-only-based group [42]. In our institution, the application of cyclophosphamide was largely omitted in favor of fludarabine in combination with 8 Gy TBI.

About one third (*n* = 91, 31.9%) of the patients in the present cohort were diagnosed with ALL. Despite the assumptive paradigm shift towards chemotherapy-only conditioning, there may be a larger impact of TBI-based conditioning regimens on lymphoblastic neoplasms. Therefore, the advantage of TBI-based conditioning in ALL appears to be more relevant and is supported by more robust data [43–46]. In this regard, a recent multinational, randomized trial has shown the superiority of total body irradiation plus etoposide compared with chemotherapy-based conditioning in childhood ALL [47]. Grün et al. further reported on long-term outcome, toxicities, and secondary malignancies in a cohort of 109 children and adolescents treated with 12 Gy TBI predominantly for ALL. In this recent analysis, the authors confirmed TBI-based conditioning regimens as feasible and effective treatment modality in the management of childhood leukemia. A broad but manageable spectrum of long-term toxicities occurred in 43% of all patients, while secondary malignancies (*n* = 3, 2.8%) represent a so far rare event but require a close follow-up [48].

Particularly in childhood and in younger adults, TBI-based conditioning is an evident risk factor for long-term complications and secondary malignancies [9, 49, 50].

Nevertheless, the risk of secondary malignancies in adults is assumed to be considerably lower. In the present study, 35 (12.3%) secondary malignancies occurred in 33 individuals after a median time of 5.8 years (70 months). Seven patients died as a consequence of the secondary malignancy. These findings are comparable to earlier reports [8–10, 15]. Furthermore, our study showed that patients diagnosed with AML or other myeloid diseases had also an increased risk of secondary malignancies (*p* = 0.048) compared to patients with ALL or high-grade lymphomas. These findings are previously described by Bhatia and others, who similarly reported a higher incidence of secondary malignancies in patients with myeloid neoplasms [51].

Intriguingly, there was an inverse correlation between total dose and the incidence of secondary malignancies. In a recent, comprehensive analysis among 4905 allo-HSCT survivors performed at the Fred Hutchinson Cancer Research Center in Seattle, the investigators demonstrated a strong effect of TBI dose and dose fractionation on the risk of the development of secondary malignancies. Patients exposed to single fraction TBI (6–10 Gy) had the highest standardized incidence ratio of secondary malignancies [50]. Fractionation could lower this risk significantly, while excessive dose escalation (13.2–17.5 Gy) offsets this effect. Moreover, for intermediate doses the risk of secondary malignancies was slightly attenuated but still significantly increased compared with low dose TBI (2–4 Gy) or chemotherapy-based conditioning regimes [50]. Of note, no significant difference was reported after low-dose TBI compared with chemotherapy-only conditioning regimens. Ultimately, these results verify a strong dose- and fractionation-dependent effect of total body irradiation as a major risk factor for secondary malignancies [52]. In our study, the majority of patients received an intermediate dose with 8 or 12 Gy TBI. Only a minority were treated with low-dose TBI (4 Gy) and are therefore underrepresented. In a considerable number of patients, lower doses were administered due to complications, while higher doses were initially prescribed. Nevertheless, there was no extreme dose gradient. In addition, lower doses were most commonly prescribed for AML patients who are at increased baseline-risk for developing secondary malignancies compared to ALL patients (8 vs. 12 Gy). These could be two explanations for the inverse relationship between the applied dose and the occurrence of secondary malignancies, in contrast to the anticipated and suggestive positive relationship. These results stress the fact that the applied radiotherapy dose might only play a subordinate role in the occurrence of secondary malignancies.

In terms of further toxicity, we have reported numerous nonmalignant sequela. Pulmonary and renal toxicity was more prevalent in individuals receiving a lower dose, which might be partially explained by the use of individual lung shields for doses beyond 8 Gy highlighting its importance [53]. However, other explanations, such as an increased age or fludarabine-based chemotherapy regimens in patients with lower TBI dose, may contribute to pulmonary toxicity and further disguise the impact of radiation dose and lung shielding. In this context, Oertel et al. have presented two recent analyses focusing on long-term toxicities after TBI [54, 55]. In partial concordance with our results, they could not detect any difference in pulmonary toxicity after 8 or 12 Gy TBI, highlighting the complex interplay of various factors influencing toxicity [54]. In addition, the authors were able to provide a comprehensive analysis of long-term sequelae in a large cohort of patients who underwent TBI demonstrating a significantly higher rate of ocular toxicities (*p* = 0.013) and severe mucositis (*p* < 0.001) in patients treated with 12 Gy TBI. No higher rate of other side effects was observed in the 12 Gy group, consistent with our findings [55].

There are several limitations that are not exclusive to the retrospective analysis of this single-center experience. We are not able to report all secondary malignancies, while no clear distinction was made between posttransplant lymphoproliferative disorders (PTLD), hematologic and solid malignancies. Moreover, some of the documentation of long-term toxicities is certainly incomplete. With a median follow-up of only 102 months, there will be more events to report in future. Chronic GVHD is a major risk factor for skin cancer [56], which may be considered outside this general analysis. In addition, patients’ personal risk behavior, such as smoking and alcohol consumption, could not be recorded [57, 58]. Surviving allo-HSCT should result in an extensive posttransplant cancer screening anyway [59, 60]. Furthermore, numerous conditioning regimens and protocols were applied during our observation period leading to a heterogeneous cohort. The different chemotherapy regimens will have contributed significantly to survival and toxicities, although we did not include this in our analysis. The inclusion of discontinued TBI series may have further biased the results. We must emphasize that we cannot draw a final conclusion and it is not possible to prove any dose dependency, reflecting the complex interplay of numerous factors influencing outcome and toxicity. On the other hand, within the dose range used in our cohort, there appears to be a correlation between a higher TBI dose and disease-specific survival. However, it seems highly unlikely that a higher TBI dose has a beneficial effect on survival in the intermediate dose ranges, and these results are subject to confounding and substantial bias. No decisive and critical difference in long-term toxicity was observed when comparing 8 Gy and 12 Gy TBI concepts. In summary, lower treatment-related mortality has led to significantly improved overall survival, but posttransplant relapse remains the most common cause of treatment failure and death in the last year. Still, TBI continues to be an integral part of many treatment concepts and should be further pursued. Balancing treatment-related mortality and disease-specific survival remains a critical consideration in finding an optimal and individualized conditioning regimen, as long-term toxicities continue to impact quality of life.

## Supplementary Information

**Supplemental Table 1: **Subgroup analysis of the occurrence of adverse events and sequelae according to the median dose applied to all patients (10.5 Gy). Patients who received low-dose total body irradiation (TBI) or have not completed TBI were excluded, assuming their poor performance status. Significant values are given in *bold*

**Supplemental Table 2 **Occurring disorders in health after treatment depending on the total dose for all patients
