# Supplementary material for: Twenty years of experience of a tertiary cancer center in total body irradiation with focus on oncological outcome and secondary malignancies
Source: Strahlenther Onkol. 2022 Mar 22;198(6):547–57. doi: 10.1007/s00066-022-01914-5 (PMC9165288; doi:10.1007/s00066-022-01914-5)
Supplement: Supplementary file 1 — Supplemental Table 1: Subgroup analysis of the occurrence of adverse events and sequelae according to the median dose applied to all patients (10.5 Gy). Patients who received low-dose total body irradiation (TBI) or have not completed TBI were excluded, assuming their poor performance status. Significant values are given in bold [file 66_2022_1914_MOESM1_ESM.docx]

|  | **n Number of patients*** | **Dose ≤ 10.5 Gy** | **Dose > 10.5 Gy** | **p** |
| --- | --- | --- | --- | --- |
| **Relapse** |  |  |  |  |
| No  Yes | 166 (69.7%)  72 (30.3%) | 67 (40.4%)  29 (40.3%) | 99 (59.6%)  43 (59.7%) | 0.990 |
| **Secondary Malignancy** |  |  |  |  |
| No  Yes | 206 (86.6%)  32 (13.4%) | 79 (38.3%)  17 (53.1%) | 127 (61.7%)  15 (46.9%) | 0.184 |
| **Cardiac Disease** |  |  |  |  |
| No  Yes | 215 (90.3%)  23 (9.7%) | 84 (39.1%)  12 (52.2%) | 131 (60.9%)  11 (47.8%) | 0.225 |
| **Pulmonary Disease** |  |  |  |  |
| No  Yes | 208 (87.4%)  30 (12.6%) | 76 (36.5%)  20 (66.7%) | 132 (63.5%)  10 (33.3%) | **0.002** |
| **Hepatic Disease** |  |  |  |  |
| No  Yes | 237 (99.6%)  1 (0.4%) | 95 (40.1%))  1 (100%) | 142 (59.9%)  0 | 0.225 |
| **Renal Damage** |  |  |  |  |
| No  Yes | 163 (68.5%)  75 (31.5%) | 55 (33.7%)  41 (54.7%) | 108 (66.3%)  34 (45.3%) | **0.002** |
| **Endocrine Disorders** |  |  |  |  |
| No  Yes | 206 (86.6%)  32 (13.4%) | 81 (39.3%)  15 (46.9%) | 125 (60.7%)  17 (53.1%) | 0.420 |
| **Cataract** |  |  |  |  |
| No  Yes | 216 (90.8)  22 (9.2%) | 86 (39.8%)  10 (45.5%) | 130 (60.2%)  12 (54.5%) | 0.609 |
| **Acute Toxicity** |  |  |  |  |
| No  Yes | 162 (68.1%)  76 (31.9%) | 64 (39.5%)  32 (42.1%) | 98 (60.5%)  44 (57.9%) | 0.705 |
