# Supplementary material for: Twenty years of experience of a tertiary cancer center in total body irradiation with focus on oncological outcome and secondary malignancies
Source: Strahlenther Onkol. 2022 Mar 22;198(6):547–57. doi: 10.1007/s00066-022-01914-5 (PMC9165288; doi:10.1007/s00066-022-01914-5)
Supplement: Supplementary file 2 — Supplemental Table 2 Occurring disorders in health after treatment depending on the total dose for all patients [file 66_2022_1914_MOESM2_ESM.docx]

|  | **≤ 8 Gy** | **> 8 Gy** | **p** | **< 10 Gy** | **≥ 10 Gy** | **p** | **<12 Gy** | **≥ 12 Gy** | **p** |
| --- | --- | --- | --- | --- | --- | --- | --- | --- | --- |
| **Recurrence**  No  Yes | 93  44 | 103  45 | 0.756 | 96  45 | 100  44 | 0.805 | 112  50 | 84  39 | 0.880 |
| **Secondary Malignancy**  No  Yes | 116  21 | 134  14 | 0.132 | 120  21 | 130  14 | 0.185 | 140  22 | 110  13 | 0.445 |
| **Cardiac disease**  No  Yes | 120  17 | 136  12 | 0.232 | 123  18 | 133  11 | 0.153 | 143  19 | 113  10 | 0.321 |
| **Pulmonal disease**  No  Yes | 110  27 | 137  11 | **0.002** | 113  28 | 134  10 | **0.001** | 132  30 | 115  8 | **0.003** |
| **Hepatic disease**  No  Yes | 136  1 | 148  0 | 0.299 | 140  1 | 144  0 | 0.313 | 161  1 | 123  0 | 0.385 |
| **Renal damage**  No  Yes | 78  59 | 113  35 | **< 0.001** | 81  60 | 110  34 | **0.001** | 102  60 | 89  34 | 0.095 |
| **Endocrine disruptions**  No  Yes | 113  24 | 131  17 | 0.148 | 117  24 | 127  17 | 0.211 | 136  26 | 108  15 | 0.360 |
| **Cataract**  No  Yes | 122  15 | 136  12 | 0.415 | 126  15 | 132  12 | 0.508 | 145  17 | 113  10 | 0.502 |
| **Acute toxicity**  No  Yes | 92  45 | 101  47 | 0.845 | 95  46 | 98  46 | 0.903 | 110  52 | 83  40 | 0.940 |
